# Effects of Customized Progressive Addition Lenses vs. Single Vision Lenses on Myopia Progression in Children with Esophoria: A Randomized Clinical Trial

**DOI:** 10.1155/2022/9972761

**Published:** 2022-02-27

**Authors:** Xiaowei Zhu, Dongmei Wang, Naiyang Li, Feng Zhao

**Affiliations:** ^1^Department of Ophthalmology, First Affiliated Hospital of Medical College of Jinan University, Guangzhou, Guangdong, China; ^2^Department of Ophthalmology, Zhongshan City People's Hospital, Zhongshan, Guangdong, China; ^3^Guangdong Provincial People's Hospital, Guangdong Academy of Medical Sciences, Guangzhou, Guangdong, China; ^4^State Key Laboratory of Ophthalmology, Zhongshan Ophthalmic Center, Sun Yat-sen University, Guangzhou, Guangdong, China

## Abstract

**Purpose:**

To evaluate the effect of customized progressive addition lenses (CPALs) versus single vision lenses (SVLs) on the progression of juvenile-onset myopia in children with near esophoria.

**Methods:**

Ninety-three Chinese children, aged 7–14 years with spherical equivalent refraction (SER) ranging from −0.50 to −4.00 D and near esophoria ≥2Δ, were randomly assigned into a CPALs (*n* = 46) and an SVLs group (*n* = 47) for a 2-year, double-masked, randomized trial. The primary outcome measure was the progression of myopia, as determined by cycloplegic autorefraction. A customized near addition, calculated by a regression equation, was prescribed to establish a fixed heterophoria status for each child, which was −3^Δ^ exophoria.

**Results:**

Eighty-four (90.3%) of the 93 children completed the 2-year follow-up. The mean initial near addition lenses were 1.65 ± 0.07 D (mean ± SE). The adjusted 2-year myopia progression was 0.23 ± 0.08 D slower in the CPALs group than in the SVLs group (*p*=0.046). Post hoc analysis found significantly larger treatment effects for CPALs in children without myopic parents (0.47 ± 0.15 D; 95% CI: 0.18–0.76), with lower baseline myopia (0.33 ± 0.09 D; 95% CI: 0.14–0.52; *p* < 0.05), with higher baseline accommodative lag (0.36 ± 0.11 D; 95% CI: 0.12–0.60; *p* < 0.05), and with higher baseline near esophoria (0.30 ± 0.10 D; 95% CI: 0.12–0.48; *p* < 0.05).

**Conclusion:**

CPALs exerted a significant but minimal protective effect against myopia progression in Chinese children with esophoric myopia, as compared with SVLs. Regulating near heterophoria and accommodative lag by near addition lenses may not be an appropriate way to prevent myopia progression.

## 1. Introduction

Chinese teenagers are susceptible to myopia leading to high prevalence and incidence in China [1–3]. This places a vast annual economic burden in terms of changing spectacles and other myopia control methods. In addition, myopes are subject to significant public and occupational health burdens, such as vision loss due to glaucoma, myopic macular hole, and myopic retinal detachment [4].

Numerous studies have claimed that myopia is associated with near work [5–7], which is usually accompanied by accommodative lag [8], a primary potential cause to myopia, for which keep the eye hyperopia defocused. Progressive addition lenses (PALs) may control the progression of myopia by decreasing the accommodative lag during near work in myopes. Leung and Brown [9] observed that when Chinese children with myopia wore PALs, both their refraction and axial lengths increased less than those of age-matched children wearing single vision lenses (SVLs). However, some well-designed studies failed to show definite benefits of PAL wear on myopia progression. The Correction of Myopia Evaluation Trial (COMET) reported a 0.20 D difference between PALs and SVLs groups at a 3-year visit, which was statistically but not clinically significant [10]. Yang et al. also observed only a statistically significant difference in the progression of myopia between a PAL group, wearing +1.50-D near addition lenses, and an SVL group, in a study of 240 Chinese children aged 7–13 years [11]. However, the study found a significant difference between treatment groups in children with esophoria in 2 years (0.77 D; *p* < 0.01). Similarly, the COMET study found that myopes with esophoria in combination with a large accommodative lag and some other related factors experienced a more marked treatment effect from PALs [12, 13]. The COMET2 study specifically enrolled children with esophoria who had large accommodative lags and found a difference of 0.28 D within a 3-year period [14].

It is possible that using PALs during near work decreases accommodative lag and esophoria in children with myopia. However, myopic children with esophoria using the same near addition lenses may become exophoric or orthophoric, or may remain esophoric during near work. If the near heterophoria participates in the progression of myopia, correcting near heterophoria with customized progressive addition lenses to a fixed value may exert a better effect on myopia children with near esophoria. However, this hypothesis is not well investigated yet.

Previous studies [11, 12] have reported that PALs may have a more marked effect in myopic children with near esophoria and high accommodative lag. Near addition lenses can simultaneously decrease accommodative lag and esophoria; however, which of these plays the major role is unclear. In our previous study [15], we reported that there was insufficient evidence to conclude that near lag provided a stimulus to myopic progression; using the same near addition lens for all myopic children with different degrees of esophoria may be inappropriate. And there is a controversial relationship between myopia and heterophoria due to the lack of prospective clinical trials to elucidate their relationship.

Following our previous study [11], we designed a 2-year prospective, randomized, double-masked clinical trial (customized progressive addition lenses (CPALs) study) wherein esophoria associated with myopia was corrected to fixed heterophoria, with an associated decrease in measured accommodative lag. It is expected to investigate the underlying mechanism of accommodative lag and near heterophoria to myopia in myopia children with esophoria and provide new evidence to control myopia progression using PALs.

## 2. Methods

All experimental protocols and procedures adhered to the tenets of the Declaration of Helsinki and were approved by the Ethics Committee of Zhongshan Ophthalmic Center, China. This study has registered on http://www.chictr.org.cn/showproj.aspx?proj=26059. Consent was obtained from the enrolled children and their parents, after providing them with a verbal and written explanation of the nature and possible consequences of the clinical trial.

### 2.1. Estimation of the Sample Size

Based on our previous data, a mean increase in myopia of approximately 1.50 D was estimated in the SVLs group over 2 years, and an increase of 0.75 D, half of that in the SVLs group, was anticipated as a clinically significant effect for the PALs group. Assuming a value of 0.75 D as the standard deviation (SD) of the difference in the change of refractive error between the two groups, we calculated that 27 participants were needed in each group for an alpha level of 0.01 and 90% power. Given the possibility of dropout, which was set at a maximum rate of 20%, we aimed to enroll at least 33 participants per group.

### 2.2. Participant Selection

Participants were included if they met the following inclusion criteria: a spherical equivalent refractive error (SER), that is, the sum of the spherical error and half the cylinder error, of −0.75 to −4.00 D (measured under cycloplegia); astigmatism not exceeding 1.50 D; anisometropia not exceeding 1.00 D in spherical or cylindrical error; best-corrected visual acuity of at least 6/6; near phoria (Δ) ≥2^Δ^ (measured by the cover test with prism neutralization with the spectacles best-corrected distant vision); absence of ocular conditions that might affect refractive development; no prior use of bifocal or PALs; and no experience of or intention to wear contact lenses. Participants also had to be aged 7–14 years at recruitment; have had a birthweight of more than 1250 g; be willing to wear glasses constantly; be available for follow-up for at least 2 years; and have no systemic conditions or ongoing medications that affect refractive development. Additionally, the participants' parents had to understand and accept that their child would be randomly assigned to either of the two spectacle lens groups. Participants were randomly assigned into each group by a random number table.

### 2.3. Recruitment and Follow-Up

All participants were recruited in the outpatient clinic. Families were informed verbally about the nature of the study and about random allocation to a particular treatment group. Once they agreed to participate by giving written informed consent, children were examined to confirm that they met the enrollment criteria. Each child was then allocated to either the CPALs or the SVLs group according to a predetermined random sequence. Parental refractive errors were also determined by noncycloplegic autorefraction. The participating children were then classified into categories with myopic parents (one or both myopic parents) or without myopic parents, with myopia defined as an average SER of less than −0.75 D for both eyes.

**Figure 1:**
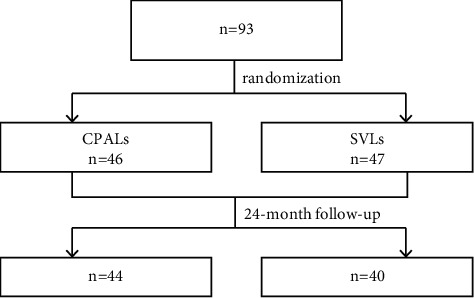
Study flow and random assignment of participants. CPALs, customized progressive addition lenses; SVLs, single vision lenses.

After enrollment, the children were followed up every 6 months for the next 2 years. At each visit, the refraction, axial length, horizontal heterophoria, and accommodative response of the participants were examined. Spectacle prescription was determined by subjective refraction, with an end point of the minimum minus dioptric power for best visual acuity. The first criterion used for a change of spectacles was myopia progression greater than 0.50 D or as per the clinical indication. Throughout the study, every measurement was taken by the same examiners masked to the treatment assignment using the same instrument, to decrease personnel and instrument bias.  Figure 1 shows the study flow and the random assignment of participants. During every two visits, a masked staff would do a telephone follow-up to teach the parents and children to correctly use glasses and asked if there is any discomfort or other questions. When necessary, the staff would report the problems to a consulting ophthalmologist who was not masked.

### 2.4. Intervention

The children were provided with either SVLs (Sola Optical, Guangzhou, China) for the SVLs group or PALs (Sola Optical, Guangzhou, China) with a short corridor (10 mm) for the CPALs group. All children were taught to use the near addition part during near work, which required special head and eye postures (head up and eyes lowered). The near addition power for each wearer was determined individually. Our previous study showed that the amount of esophoria and accommodative lag in juvenile esophoric myopes was negatively correlated with an increase in the power of the near addition [16]. As we found that the amount of near phoria and accommodative lag could not reach a minimum value with the same power of the near addition, we adjusted the power of the near addition to that of the near phoria considered ideal for the PAL in order to arrest myopia progression in children with esophoria in the current study.

**Figure 2:**
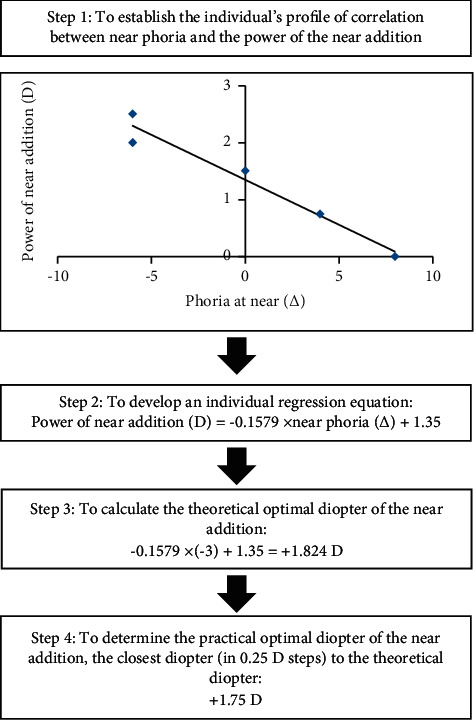
The procedures for determining the optimal near addition.

The fixed phoria status defined in the present study was −3^Δ^, as suggested by a recent study [17, 18]. Near phoria for each participant was measured at 33 cm, with their distant prescription to watch an E target (20/32 letter) in place combined with 0 D, +0.75 D, +1.50 D, +2.00 D, or +2.50 D near addition in sequential order. To adapt to the new near prescription, phoria measurement was performed only after 10 min of reading [19]. Thereafter, a regression equation was developed for each participant. Then, the optimal near addition (e.g., the one that was closest to the value calculated from the equation) was selected.  Figure 2 shows the procedure to determine the optimal near addition for one random sample. The optimal near addition power was adjusted at each visit, according to the participants' latest phoria status and refractive error, which was the second criterion for a change of spectacles.

### 2.5. Outcome Measurements

At each visit, the objective refractive error was measured by cycloplegic autorefraction (Topcon AR 8800, Tokyo, Japan), which was performed 30 min after the third administration (with administrations spaced at 5-min intervals) of Mydrin®-P (0.5% tropicamide + 0.5% phenylephrine hydrochloride; Santen Pharmaceutical Co., Ltd., Osaka, Japan), which has been proven to be an acceptable and useful cycloplegic agent in Asian school children with a wide range of myopic refractive errors [20]. The average of five reliable readings was taken as the objective refractive error and was expressed as the SER. For each eye, the mean of five spherical equivalent autorefraction measurements was calculated. And the distance prescription was determined by subjective refraction according to the principle of maximum plus to maximum visual acuity (MPMVA).

The axial length was chosen as a metric for evaluating the change in ocular biometry because it is known to be strongly correlated with myopia progression. At each visit, the axial length was determined by coherence interferometry (IOLMaster; Carl Zeiss Meditec, Oberkochen, Germany) under cycloplegia in a dark room (<10 lux). The participants were asked to fixate forward during testing. Readings were accepted when the deviation between two sequential readings was less than 0.02 mm, and the average of five reliable readings in each eye was taken for analysis.

To monitor the possible effect of progressive lenses on heterophoria, horizontal phoria was measured at each visit. An 20/32 letter was placed at 33 cm for the near measurements and at 5.0 m for the far measurements. A prism was used to neutralize the participant's eye movements.

The accommodative response was measured using an open-field autorefractor (SRW-5001K; Shin-Nippon, Tokyo, Japan) [21]. Participants, with a trial frame fitted with the distance prescription in place with 0 D, +0.75 D, +1.50 D, +2.00 D, and +2.50 D addition lenses in sequential order, were instructed to fixate binocularly at near (33 cm, on a 5 × 5 array of “E” targets in N10 size) and keep fixation clear. The average of three readings was used to calculate the accommodative response according to the formula used by Gwiazda and colleagues [22].

### 2.6. Masking and Compliance

The examiners (optometrists) collecting the biometry data (refractive error, axial length, etc.) or prescribing spectacles were masked to the treatment assignment. All the parents and children were encouraged to use glasses in the same way as the guidelines for PALs and were asked to refrain from discussing any issues related to the types of study glasses with the masked examiners. A masked staff who was responsible for telephone follow-up, calling the parents and children return visit, and collecting the glasses before examination. A consulting ophthalmologist who did not take part in collecting, recording, and analyzing data was responsible for the group allocation, frame selection, appropriately providing spectacles with CPALs or SVLs, measuring the aided visual acuity on arrival, checking and adjusting the fit of the spectacles, checking compliance, responding to questions, dealing with nontolerance cases, and the accuracy of data entry.

Compliance with and adherence to the assigned spectacles were assessed at every follow-up visit by means of a questionnaire filled out by both the children and their parents. The questionnaire was adapted for this study from the one used by Saw [23] and included the total time of spectacle wearing (three options for the duration of spectacle wear: constantly, no less than half of the awake time per day, or less than half of the awake time per day) and whether the children viewed near objects through the lower part of the lens. Only children who reported constantly wearing the spectacles and viewing objects through the correct area of the spectacle lens were considered to have good compliance and adherence.

### 2.7. Statistical Analyses

All statistical analyses were carried out independently by an experienced statistician using SPSS v16.0 software (SPSS Inc., Chicago, IL, USA). Study results are expressed as mean ± standard error of mean (SE). Baseline characteristics were compared between the groups using unpaired, two-tailed *t*-tests, if normality assumptions were met, or using Wilcoxon's rank-sum test for continuous data, and the chi-square test for categorical data. Subgroup analyses of the treatment effect were also conducted to examine the influence of the baseline covariates such as myopia in parents, accommodative lag, baseline SER, or age, and then, a mixed-model, two-way analysis of variance (ANOVA) was used to determine whether there was an interaction between the covariates and the treatments. The significance level for all tests was set at *p* < 0.05.

## 3. Results

From July to August 2012, 93 children were enrolled in this study, with 46 in the CPALs group and 47 in the SVLs group. Clinical characteristics at baseline were comparable between the two groups, with no statistically significant or clinically relevant differences (Table 1). All children adapted to the study glasses successfully. Similar to previous reports, the questionnaire survey administered at each scheduled visit identified no adverse effects associated with using CPALs (questionnaire survey of spectacle wearability, safety, and compliance in myopic children; PALs versus single focus lenses) except for a transient uncomfortable feeling in several children at the very beginning of the wearing period.

Two-year retention was excellent, with only nine children, two in the CPALs and seven in the SVLs groups, who failed to return for the final visit and were excluded from the analysis. The reasons for being lost to follow-up included incompatibility with cycloplegic eye drops (one in the SVLs group), relocation to other prefectures (three in the SVLs group and one in the CPALs group), and a preference for wearing contact lenses (three in the SVLs group and one in the CPALs group). After excluding the nine candidates who dropped out, clinical characteristics at baseline remained balanced between the two groups. Of the nine dropouts, two in the CPALs group and six in the SVLs group had completed a 6-month follow-up. The progression of myopia in 6 months was 0.31 ± 0.21 D in the CPALs group, and 0.42 ± 0.23 D in the SVLs group. There was no statistically significant difference between the two groups. Self-reported compliance and adherence to spectacle wear were good, as assessed by the answers to the questionnaires provided by both the children and their parents. Of the 84 participants, 74 (88%) reported constant wearing of their glasses and viewing through the appropriate areas of the lenses.

### 3.1. Change in the Refractive Error between Groups

**Figure 3:**
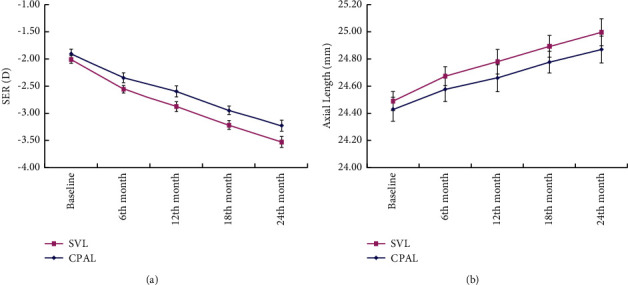
(a) Mean cycloplegic autorefraction (spherical equivalent refraction, SER) and (b) mean axial length for both the groups at each visit. Error bars: ±1SE.

Since there was a high correlation between the SER of both eyes in an individual (*R*^2^ = 0.86, *p*=0.014), the average SER of the two eyes was used for analysis. At the baseline, there was no significant difference in the SER between the two groups. The change in the mean SER over 2 years is shown in Figure 3(a). The 2-year myopia progression adjusted for age, gender, and baseline covariates including SER, accommodative lag, number of myopic parents, and near phoria was −1.32 ± 0.08 D in the CPALs group and −1.55 ± 0.09 D in the SVLs group, respectively, which was statistically significant for both the groups (paired *t*-tests: *p* < 0.001). The intergroup difference of 0.23 ± 0.08 D was also statistically significant (unpaired *t*-test: *p*=0.046), which was less than the clinical difference of 0.75 D. The annual year myopia progressions were -0.66 ± 0.11 D vs −0.82 ± 0.12 D for the first year (*p* < 0.05) and −0.66 ± 0.09 vs −0.73 ± 0.11 D for the second year (*p* > 0.05).

### 3.2. Progression of Myopia by Baseline Characteristics

Table 2 presents the 2-year change in the SER for both the groups and the corresponding mean changes for each baseline characteristic. Significant differences between both the groups were observed in children without myopic parents, with lower baseline myopia, with higher baseline accommodative lag and with higher baseline near esophoria (*p* < 0.05). The data of baseline myopia subgroups were analyzed ulteriorly. The lower baseline myopia subgroup was defined as less than −2.00D, with -1.38 ± 0.06D (−1.00 D∼−2.00 D) and with 4.86 ± 0.31^Δ^ near phoria (2 ^Δ^ ∼ 8 ^Δ^). The higher baseline myopia subgroup was defined as more than −2.00D, with −2.80 ± 0.11D (−2.00 D∼−4.00 D) and with 6.07 ± 0.47^Δ^ near phoria (2 ^Δ^∼14 ^Δ^). The myopia progression of the lower and higher baseline subgroup was 1.30 ± 0.15 and 1.62 ± 0.16 with significant difference (*p* < 0.05). But the near additions of the lower and higher baseline subgroup were 1.71 ± 0.11 D and 1.79 ± 0.13 D without significant difference (*p* > 0.05).

**Figure 4:**
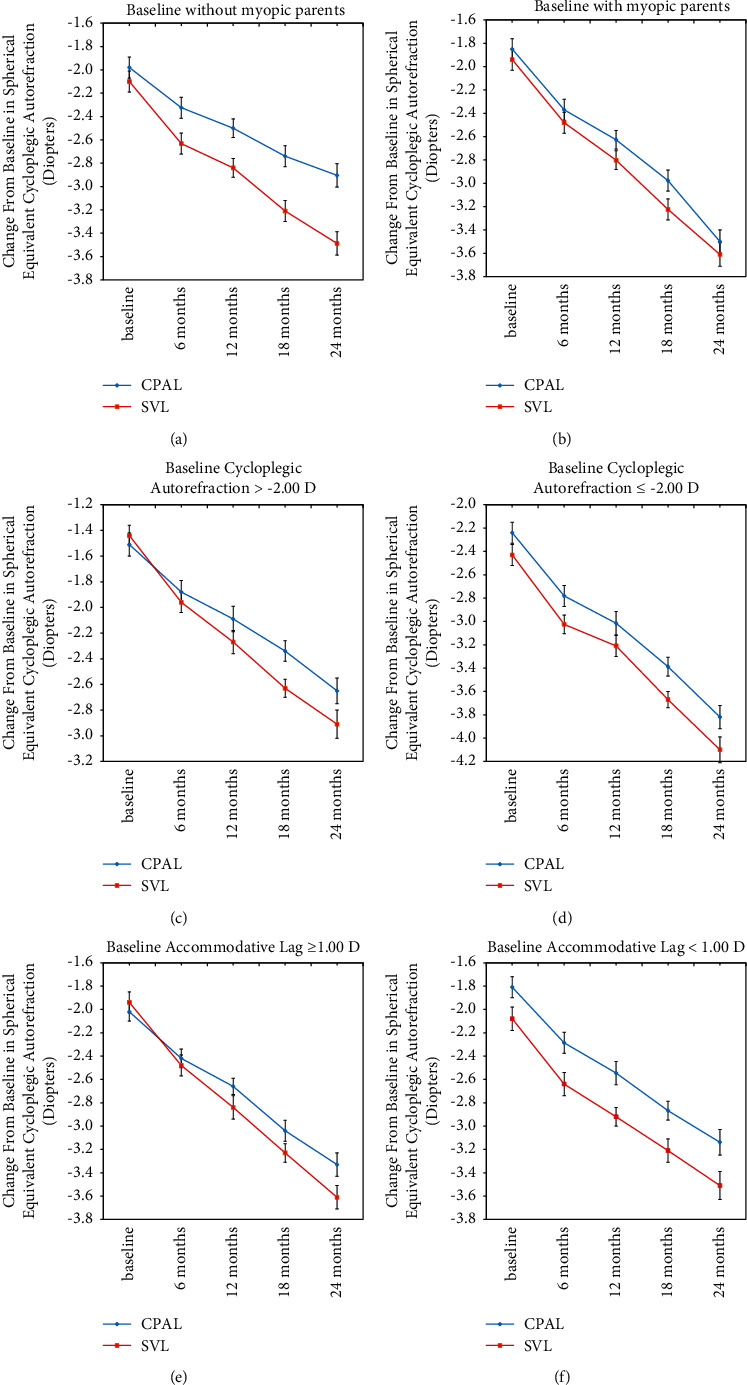
Mean progression of myopia in the CPALs and SVLs groups for three of the covariates, without/with myopic parents (a, b), baseline myopia (c, d), and baseline near accommodative lag (e, f). Error bars: ±1 SE.

Interaction analysis was then conducted to identify whether the treatment effect differed in subgroups defined by any of the baseline characteristics (e.g., in children with myopic parents versus those without myopic parents). We observed that a significant interaction between the treatment effect and parental myopia, with the treatment found to be more effective by 0.45 D (*p*=0.021) in children without myopic parents compared to those with myopic parents. There was also a significant interaction between the treatment and baseline myopia, with the treatment being more effective by 0.24 D (*p*=0.047) in children with lower baseline myopia than in those with the higher baseline myopia. Furthermore, there was a significant interaction between the treatment and the baseline accommodative lag, with the treatment being more effective by 0.26 D (*p*=0.039) in children with higher baseline accommodative lag than in those with lower baseline accommodative lag. The unadjusted mean progression of myopia against the covariates showing significant interaction with the treatment is plotted in Figure 4.

### 3.3. Changes in Customized Addition Lenses, Near Phoria, and Accommodative Lag after Treatment

The initial power of the customized near addition was 1.65 ± 0.07 D (ranged from 1.00 to 2.75 D). This was followed by 1.71 ± 0.07 D, 1.84 ± 0.08 D, and 1.86 ± 0.07 D at 6, 12, and 18 months (ranged from 1.25 to 2.75D), respectively. There was no statistically significant difference between these time points (one-way ANOVA, *p*=0.67). Esophoria at 33 cm was 4.81 ± 0.37^Δ^ (ranged from 2 to 10^Δ^) and 5.84 ± 0.51^Δ^ (ranged from 2 to 14^Δ^) in the SVLs and CPALs groups. After 2 years of treatment, near esophoria changed slightly in both the groups (2.12 ± 0.45^Δ^ in the CPALs group and 1.76 ± 0.48^Δ^ in the SVLs group), without statistical significance. Accommodative lags at 33 cm with prescriptions for distant correction changed slightly (0.28 ± 0.13 D in the CPALs group and 0.15 ± 0.11 D in the SVLs group), once again without a significant difference.

### 3.4. Changes in the Axial Lengths between Groups

The mean increase of the axial length was 0.68 ± 0.05 mm in the SVLs group and 0.61 ± 0.04 mm in the CPALs group, and this increase seen in both the groups was statistically significant (paired *t*-tests, *p* < 0.001). Mean changes in the axial length correlated significantly with those in the refractive error (*R*^2^ = 0.85 for SVLs and *R*^2^ = 0.87 for CPALs). There was a statistically significant difference in the increase of the axial lengths between the two groups (unpaired *t*-test: *p*=0.021). The mean axial length in the two groups over the 2-year period is shown in Figure 3(b).

### 3.5. Visual Burden

The age of myopia onset was 8.79 ± 0.45 yrs in the SVLs group and 9.23 ± 0.33 yrs in the CPALs group without significant difference. Participants spent 6.3 ± 0.69 and 6.8 ± 0.82 h/day (*p* > 0.05) in the SVLs and CPALs groups on near work, respectively. There was no significant difference in outdoor activity between the two groups, with 10 ± 1.9 and 12 ± 1.5 h/week in the SVLs and CPALs groups, respectively.

## 4. Discussion

The prospective, randomized, double-masked study evaluated the effectiveness of the new design of CPALs on myopia progression in Chinese children with near esophoria and myopia in which near additions were periodically adjusted based on the equation calculated by the near esophoria at every visit. CPALs resulted in a significant but weak treatment effect in myopia progression of 0.23 D after 2 years of treatment. The control effect was obviously weaker than nonsingle vision contact lenses, Ortho-K lenses or atropine. This study showed that corrected near phoria state with near addition lenses was not clinically beneficial for esophoria myopia children, implying that accommodation and heterophoria are related to myopia, but not the primary factors to regulate the progression of myopia.

Numerous studies showed the contradictive results of accommodative lag to the progression of myopia. However, the treatment effect of CPALs was significantly greater in the subgroup with a larger accommodation lag than in the subgroup with smaller accommodation lag at baseline. These results seem to be consistent with previous clinical trials using bifocals [24] or PALs [10, 12]. However, several studies showed that, irrespective of whether accommodative lag was large or small, there was no association between accommodation lag and myopia progression [15, 25, 26]. There are two hypotheses explaining the contradiction. First, accommodative errors can be measured subjectively or objectively. The objective test of accommodative errors used in the present study provided an overestimation as compared to subjective measurement [27, 28], which may be due to an inappropriate method [29]. Moreover, objective accommodative errors cannot be compared across different studies, due to different tests influencing factors, such as pupil size and light environment [30, 31]. Second, Lan [15] and CLEERL [25] studies provided direct evidence that baseline accommodative lag did not correlate with myopia progression. On the other hand, the present study and Gwiazda et al.'s study [12] provided indirect evidence that accommodative lag was involved in myopia progression, as myopia progression was slowed by decreasing accommodative lag. The myopia control effect could have resulted from other mechanisms.

Correcting near esophoria using CPALs did not show a better protective effect on myopia. The study's participants were specifically recruited because they had myopia with near esophoria, as in a previous study we found that a subgroup of children with near esophoria had the greatest 2-year PAL treatment effects of 0.77 D [11]. However, CPALs in this study showed fewer benefits than nonsingle vision contact lenses in controlling myopia progression with 0.29D of 1 year [32], 0.25D of 10 months [33], 0.52D of 2 years [34], and 0.57D of 1 year [35].

Moreover, epidemiological studies have found esophoria was correlated with hyperopia, whereas near exophoria was correlated with myopia [36]. And near esophoria may participate in the onset and progression of myopia [37, 38]. However, some studies demonstrated that refractive error or myopia was not associated with heterophoria [39, 40]. In consideration of near heterophoria, a clinical study revealed the treatment effect of group (1.05 D) using prismatic bifocal spectacles with 6^Δ^ base-in prism was similar to bifocal group (0.81 D) in a 3-year study [18]. Therefore, these data might prove that regulating the phoria state was not an appropriate choice for controlling myopia progression as our study showed.

Meanwhile, the practical limitations of bifocal and PAL spectacles for school-aged children and their implications for compliance cannot be ignored. Once inserted, contact lenses are more likely to be left in place throughout the day than spectacle lenses for practical reasons, thereby favoring compliance. Moreover, assuming that the maximum treatment effects of PAL spectacles require children to use the near addition during near work, children are less likely to be compliant because of the unusual head and eye postures required (head up and eyes lowered) and because of the optical distortions encountered on viewing obliquely through the lower regions of such spectacle lenses. Furthermore, with the extensive use of computers with screens at or above eye level by today's school-aged children, one could argue that the more traditional multifocal spectacle lens designs are not practical for these age groups. Children are likely to have had difficulty in looking over the top of the near segments while reading on screens. Nonsingle vision contact lenses represent the end of this continuum, with exposure to the addition power being independent on gaze direction and/or training, and with the only requirement being that the lenses are worn on a daily basis. The newest spectacle design of defocus incorporated multiple segments (DIMS) lenses showed a 51.7% myopic control rate, which was way better than that obtained in the present study. The main reason for this is that there was no restriction of spectacle use, and myopic defocus could continue to work [41].

Regional and ethnic factors may also affect the treatment of myopia. Previous studies [11, 42–44] indicated that juvenile-onset myopia progression ranged from 0.50 D to 1.00 D per year in Asia. In our study, the progression rates were 0.66 D and 0.78 D per year in the CPAL and SVL groups, respectively, which were much larger than the 0.4 D per year progression rate reported in the COMET study. It could be hypothesized that the progression of myopia in children with a family history of myopia was too rapid to treat; however, in the COMET participants, myopia could be slowed by PALs. Interestingly, the treatment effect of children without a family history of myopia was 0.47 D. The results differ from COMET studies, which claimed that PALs were more effective in the subgroup of children with myopic parents. The different results might result from the different ethnics. Compared to the COMET study, participants in the present study were Chinese, spending more time on near work and less time on outdoor activities, which were the most two important risk factors of myopia [45, 46].

Baseline myopia was deeply analyzed as an important risk factor on myopia treatment. In our study, participants showed a better treatment effect in the lower baseline myopia group compared to the higher group (0.33 vs. 0.09 D). The present study showed opposite result to previous reports with protective effect found in the higher baseline myopia group. In a Singapore orthokeratology study, axial length does not change significantly in children with baseline myopia above 4.00D in 12 months [47]. And a Chinese orthokeratology study also shows that higher baseline spherical equivalent error is associated with slower axial elongation [48]. In our study, the myopia progression turned out faster in the higher baseline myopia group, combined CPAL and SVL groups. We speculate that CPALs had such a weak effect that cannot control high-speed myopia progression. Besides, the higher baseline myopia was related to longer myopia history and earlier myopia onset age. These factors may weaken the treatment effect to some extent.

Undoubtedly, several individual variations such as the age of myopia onset, the time spent on near work, and outdoor activities could not be ignored and balanced, while analyzing myopia control effect. A study comparing the myopia progression of Finnish and Singaporean children suggested that earlier myopia onset age means more myopia progression [49]. There were no significant differences in myopia onset age between the two groups in this study. Limited by the small samples and weak effect of this study, it was meaningless to take the myopia onset age for further analyze. Several types of research had proved that myopia was closely related to more near work and less outdoor activities [50–52]. The near workload and outdoor activities showed no significant difference between the two groups as well; however, these data were collected by questionnaire with a subjective bias to some extent.

The study had some limitations. First, CPALs were mainly designed to eliminate esophoria, as previous studies showed a larger effect of PALs in myopic children with esophoria. However, according to Jiang [18], addition power associated with zero accommodative lag was larger than that associated with -3^Δ^ phoria, and thus, there might be some residual accommodative lag with a customized near addition of 1.73 ± 0.07 D. This meant that less near addition used in our study might attenuate treatment effect to esophoria myopia children. In other words, it verified that heterophoria had no or tiny effect on the progression of myopia indirectly. Second, once the children left the clinic, a questionnaire and telephone follow-up twice a year were the only two ways to monitor their spectacle wear habits at home.

In conclusion, we found that CPALs had statistically significant but no clinical treatment effect on myopia progression in Chinese children with myopia and near esophoria. Our prospective randomized double-blind research provided a reliable evidence that controlling the phoria state or accommodative lag did not significantly alleviate myopia progression. New measures according to other pathophysiological mechanisms of myopia need to be applied to slow down its progression.

## Figures and Tables

**Table 1:** Baseline characteristics (right eye only) of the participants by the study group.

Variable	CPALs group (*n* = 44)	SVLs group (*n* = 40)	Difference	*p*
Female, *n* (%)^#^	25 (57)	21 (52.5)	4 (4.5)	0.69
Age (y)^*∗*^	11.02 ± 0.24	10.75 ± 0.23	0.27	0.41
Cycloplegic autorefraction (D)^*∗*^	−1.91 ± 0.13	−2.01 ± 0.12	0.1	0.36
Axial length (mm)^*∗*^	24.43 ± 0.11	24.49 ± 0.11	−0.06	0.85
Near phoria at 33 cm (Δ)^*∗*^	5.84 ± 0.51	4.81 ± 0.37	1.03	0.08
Accommodative lag (D) at 33 cm^*∗*^	1.09 ± 0.08	1.11 ± 0.08	−0.02	0.86

^*∗*^Continuous variables, analyzed by the unpaired *t*-test. ^#^Categorical variables, analyzed by the chi-squared test.

**Table 2:** Adjusted 2-year myopia progression and mean difference between study groups defined by baseline characteristics.

Baseline characteristics	PAL group			SVL group		Difference ± SE£ (D)	Simultaneous 95% CI
	*n*	Addition lenses^#^ (D)	Mean ± SE (D)	*n*	Mean ± SE (D)		
*Gender*							
Male	19	1.76 ± 0.11	−1.21 ± 0.15	19	−1.40 ± 0.15	0.19 ± 0.11	(−0.04 to 0.42)
Female	25	1.71 ± 0.10	−1.42 ± 0.13	21	−1.67 ± 0.14	0.25 ± 0.14	(−0.03 to 0.53)

*Myopic parent* ^*∗*^^*$*^							
Without	20	1.81 ± 0.12	−0.92 ± 0.14	17	−1.39 ± 0.16	0.47 ± 0.15	(0.18 to 0.76)†
With	24	1.67 ± 0.13	−1.65 ± 0.13	23	−1.67 ± 0.14	0.02 ± 0.17	(−0.29 to 0.33)

*Age (years)*							
7∼11	20	1.69 ± 0.12	−1.45 ± 0.13	16	−1.67 ± 0.19	0.22 ± 0.13	(−0.07 to 0.52)
11∼14	24	1.77 ± 0.11	−1.21 ± 0.14	24	−1.47 ± 0.13	0.26 ± 0.10	(−0.08 to 0.61)

*Baseline accommodative lag to 3 D demand (D)* ^*$*^							
Low (<1.00)	23	1.64 ± 0.09	−1.33 ± 0.15	20	−1.43 ± 0.14	0.10 ± 0.16	(−0.25 to 0.44)
High (≥1.00)	21	1.82 ± 0.14	−1.31 ± 0.14	20	−1.67 ± 0.15	0.36 ± 0.11	(0.12 to 0.60)†

*Baseline near phoria (33 cm)*							
2< esophoria ≤6	16	1.57 ± 0.11	−1.34 ± 0.17	22	−1.50 ± 0.14	0.16 ± 0.11	(−0.13 to 0.55)
esophoria >6	28	1.82 ± 0.13	−1.31 ± 0.11	18	−1.61 ± 0.17	0.30 ± 0.10	(0.12 to 0.48)†

*Baseline cycloplegic autorefraction (D)* ^*$*^							
Less myopia (>−2.00)	26	1.68 ± 0.08	−1.14 ± 0.12	24	−1.47 ± 0.13	0.33 ± 0.09	(0.14 to 0.52)†
More myopia (≤−2.00)	18	1.80 ± 0.13	−1.58 ± 0.15	16	−1.67 ± 0.17	0.09 ± 0.12	(−0.15 to 0.33)
Overall	44	1.73 ± 0.07	−1.32 ± 0.08	40	−1.55 ± 0.09	0.23 ± 0.08	(0.04 to 0.42)†

^*∗*^SER <−0.75 D, determined by noncycloplegic autorefraction; £PAL-SVL; ^†^significant treatment effect (*p* < 0.05); ^$^significant interaction (*p* < 0.05); ^#^addition lenses were analyzed among the prescriptions of all time points.

## Data Availability

All the data used in this study are available from the corresponding author upon reasonable request.
